# Incidence and Temporal Trend in Risk Factors of Severe Infections in ANCA-Glomerulonephritis Patients

**DOI:** 10.1016/j.ekir.2020.12.037

**Published:** 2021-01-07

**Authors:** Pierre Jourdain, Benoit Brilland, Ouassim Medhioub, Jeanne Caron, Clément Samoreau, Assia Djema, Renaud Gansey, Jean-Philippe Coindre, Maud Cousin, Anne Sophie Garnier, Nicolas Henry, Samuel Wacrenier, Jeremy Riou, Giorgina Barbara Piccoli, Jean-François Augusto

**Affiliations:** 1 Service de Néphrologie-Dialyse-Transplantation, Université d’Angers, CHU Angers, Angers, France; 2 CRCINA, INSERM, Université de Nantes, Université d’Angers, Angers, France; 3 Micro et Nanomedecines Translationnelles, MINT, Université d’Angers, UMR INSERM 6021, UMR CNRS 6021, Angers, France; 4 Service de Néphrologie-Dialyse, CH de Cholet, Cholet, France; 5 Service de Néphrologie-Dialyse, CH de Laval, Laval, France; 6 Service de Néphrologie-Dialyse, CH du Mans, Le Mans, France; 7 Methodology and Biostatistics Department, Delegation to Clinical Research and Innovation, Angers University Hospital, 49100 Angers, France; 8 Department of Clinical and Biological Sciences University of Torino, Italy

Despite great improvement in anti-neutrophil cytoplasmic antibody (ANCA)−associated vasculitis (AAV) management,S1 morbidity and mortality of AAV patients remain significantly higher than in the general population. Indeed, 5-year mortality rate reaches 20% to 25%,^1^^,^^2^ mainly due to kidney and lung involvement.S2 Causes of death have been widely analyzed, and have related to vasculitis activity, cardiovascular events, cancers, and infections^1^^,^^3^^,^S1 in a complex interplay of baseline comorbidities, vasculitis activity, and regimen toxicity. Infections are not only the leading cause of death in the early phases of remission-induction treatment, but also account for substantial mortality in the long term.^4^^,^S1 Thus, the identification of subgroups of AAV patients with a higher risk of infection may be of great help in implementing preventive strategies such as dose regimen adjustment and prophylaxis with anti-infectious drugs.

Previous studies have shown that infection risk is greater within the first months of immunosuppressive treatment, and have suggested older age, renal impairment, pulmonary involvement, high disease activity, steroid dose, or lymphopenia as potential risk factors for infection in AAV patients.^5^, ^6^, ^7^, ^8^^,^S3−S5 However, to date, no study has addressed whether risk factors of infection vary over time. Thus, the aim of this study was to analyze time-dependent variations in risk factors for severe infections.

## Results

### Baseline Data

The study population included 168 patients with more than 3 months’ follow-up. In all, 144 patients (85.7%) had ANCA-glomerulonephritis (ANCA-GN) confirmed at kidney biopsy, and 24 (14.3%) had signs of kidney injury enabling ANCA-GN diagnosis. The median age at presentation was 68.0 years (range, 57.0−74.0 years), with a predominance of male patients (61.9%). Myeloperoxidase- and PR3-ANCAs were detected in 114 (67.9%) and 52 (31.0%) patients, respectively. Two patients were ANCA negative. The median Birmingham Vasculitis Activity Index (BVAS) was 16.0 (range, 12.0−20.0). The median estimated glomerular filtration rate (eGFR) was 18.1 ml/min per 1.73 m^2^*.* Remission-induction treatment consisted mainly of cyclophosphamide and steroid pulses. The median follow-up of the cohort was 47.9 (range, 18.4−93.7) months, during which 46 (27.4%) patients developed end-stage kidney disease and 43 (25.6%) died. Infections (32.5%), cardiovascular diseases (25.6%), and vasculitis (20.9%) represented the main causes of death. Death was related to cancer in 3 patients (7%), and the cause of death was not recorded in 6 patients (14%). These data are detailed in Supplementary Table S1.

### Severe Infections and Pathogens

**Figure 1:**
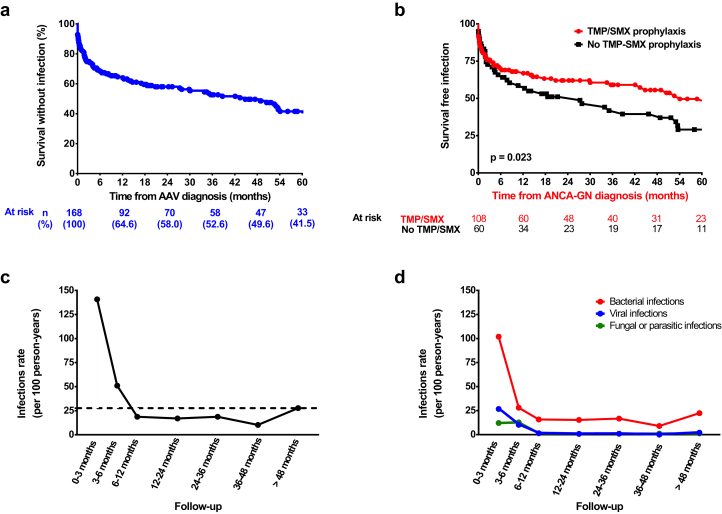
**Infectious episodes in the cohort.** (a) Survival free of severe infection and (b) survival free of severe infection according to trimethoprim−sulfamethoxazole prophylaxis. Temporal trends in infection rate, considering (c) all infection types and (d) infection subtypes (D). In (c) and (d), results are expressed using rate per 100 person-years. The dashed line represents the median rate of infection. AAV, ANCA−associated vasculitis; ANCA, anti-neutrophil cytoplasmic antibody; ANCA-GN, ANCA-glomerulonephritis.

A total of 235 severe infections developed in 90 patients (53.6%), predominantly bacterial (79.6%) and most often affecting the lung (40.6%). Of 105 positive cultures, gram-negative bacteria were the most frequently isolated germs (59%) (Supplementary Tables S2 and S3). Most patients experienced their first severe infectious episode within the first months following ANCA-GN diagnosis (Figure 1a), and 48 (25%) experienced 3 or more infectious episodes during follow-up (Figure S1). The overall infection rate was 27.7 episodes per 100 person-years: 140.8/100 person-years between diagnosis and month 3; 60.0/100 person-years between month 3 and month 6, decreasing after month 6, and remaining stable thereafter (Figure 1c and d).

### Predictors of Severe Infection

In the univariable analysis, age, intensive care unit (ICU) admission, eGFR, need for kidney replacement therapy (KRT) at diagnosis (dialysis), C-reactive protein level, lymphocyte count at ANCA-GN diagnosis, and prophylaxis with trimethoprim−sulfamethoxazole (TMP/SMX) were significantly associated with the risk of severe infection during follow-up (Table S4). In the multivariable analysis, older age (hazard ratio [HR] = 1.04, 95% confidence interval [CI] = 1.02−1.04), ICU admission (HR = 2.27, 95% CI = 1.13−4.56), and KRT at diagnosis (HR = 1.84, 95% CI = 1.01−3.35) were significantly associated with an increased risk of infection (Table 1). Treatment with TMP/SMX (HR = 0.58, 95% CI = 0.38−0.89) was associated with a decreased risk of severe infections (Table 1) and was also associated with longer survival free of severe infection (Figure 1b). Subgroup analysis showed that TMP/SMX tended to be associated with longer survival free of bacterial and fungal infection, but not viral infection (Supplementary Figure S2). Moreover, TMP/SMX prophylaxis tended to be associated with fewer *Pneumocystis jirovecii* infections (2 infected patients under TMP/SMX prophylaxis vs. 5 patients without prophylaxis at the end of follow-up, *P* = 0.098).

**Table 1:** Multivariate Cox analysis of risk factors associated with first infectious event

	Multivariate analysis of risk factor for severe infections								
	All infections			Early infections			Late infections		
	HR	CI	*P*	HR	CI	*P*	HR	CI	*P*
Baseline characteristics at ANCA-GN diagnosis									
Sex, male									
Age, yr	1.04	1.02–1.06	**<0.001**	1.04	1.01–1.07	**0.003**	1.02	1.00–1.05	0.058
BMI, kg/m^2^									
Hypertension									
Diabetes mellitus									
ANCA-associated vasculitis characteristics									
Newly diagnosed									
Admission in ICU at ANCA-GN diagnosis	2.27	1.13–4.56	**0.022**	2.24	1.07–4.69	**0.031**			
BVAS at AAV at ANCA-GN diagnosis									
MPO-ANCA or no ANCA							1.56	0.72–3.38	0.265
Organ involvement									
Cutaneous signs									
Ear, nose, throat									
Heart									
Digestive									
Lung									
Neurological									
Kidney, at ANCA-GN diagnosis									
eGFR, ml/min per 1.73 m^2^^a^	0.99	0.89–1.10	0.990	1.09	0.95–1.25	0.227	1.06	0.87–1.29	0.580
Need for kidney replacement therapy	1.84	1.01–3.35	**0.045**	2.05	0.97–4.34	0.061	1.38	0.64–2.94	0.411
Renal limited vasculitis, versus systemic vasculitis									
Biology at ANCA-GN diagnosis									
C-reactive protein, mg/l^b^									
Serum albumin, g/l									
Lymphocyte count, g/l^c^									
Immunoglobulin level, g/l^d^									
Remission-induction regimen									
Cyclophosphamide				1.85	0.62–5.49	0.270			
Rituximab									
Methylprednisolone pulses				1.59	0.61–4.11	0.337			
Plasma exchange				1.64	0.90–2.99	0.109			
Prophylaxis with TMP/SMX	0.58	0.38–0.89	**0.014**				0.61	0.35–1.06	0.081
Maintenance treatment									
Steroids at mo 6									
Steroids >10 mg/d, mo 6									
Azathioprine									
Rituximab									
Renal function									
eGFR at mo 6, ml/min per 1.73 m^2^^b^							0.80	0.64–0.99	**0.046**
Infection between ANCA-GN diagnosis and mo 6									

ANCA, anti-neutrophil cytoplasmic antibodies; BMI, body mass index; BVAS, Birmingham Vasculitis Activity Index; eGFR, estimated glomerular filtration rate; GN, glomerulonephritis; ICU, intensive care unit; MPO, myeloperoxidase; TMP/SMX, trimethoprim−sulfamethoxazole.

Bold values mean *P*-value < 0.05.

a Per 10 ml/min per 1.73 m^2^ increment.

b Per 10 mg/l increment; data available for 150 patients, excluded from multivariable model.

c Data available for 129 patients, excluded from multivariable model.

d Data available for 119 patients.

### Temporal Trend Analysis of Infection Risk Factors

Next, we performed a temporal analysis of risk factors, differentiating the first 6 months of follow-up for early infections, and after 6 months for late infections. A total of 52 patients developed a severe infection within the first 6 months, and 58 patients after 6 months.

For early infection, in the univariable analysis, risk factors were age, admission to the ICU at diagnosis, KRT need at diagnosis, and cyclophosphamide used as remission-induction regimen (Supplementary Table S4). In the multivariable analysis, only age (HR = 1.04, 95% CI = 1.01−1.07) and ICU admission at diagnosis (HR = 2.24, 95% CI = 1.07−4.69) remained significantly associated with the risk of early infection (Table 1).

For late infections, in the univariable analysis, risk factors were age, MPO-ANCAs, eGFR at diagnosis, month 6 eGFR, and TMP/SMX prophylaxis (Supplementary Table S4). In the multivariable analysis, only eGFR at 6 months was associated with late infection risk (HR = 0.80, 95% CI = 0.64−0.99). Age (HR = 1.02, 95% CI = 1.00−1.05) and TMP-SMX (HR = 0.61, 95% CI = 0.35−1.06) tended to be associated only with late infections (Table 1).

## Discussion

To the best of our knowledge, this study is the first to address the risk factors for infection in ANCA-GN patients by performing a temporal trend analysis. The major finding is that risk factors vary over time. Early infections that are associated with the induction immunosuppressive drug regimen are favored by older age and the need for ICU admission at diagnosis. Conversely, late infections are associated mainly with poorer kidney function at 6 months.

It is interesting, but not surprising, that kidney impairment was the only significant risk factor associated with late infections in the multivariable analysis, with each 10 ml/min per 1.73 m^2^ eGFR decrease conferring a 20% increase in infection risk. This suggests that renal function becomes the main risk factor for infection, more so than other classic risk factors, such as age.

Another important finding was that TMP/SMX prophylaxis was independently associated with a 40% risk reduction of severe infection. Interestingly, this effect was observed mainly for late infections and is in line with a recent study showing a 70% risk reduction of severe infections under TMP/SMX prophylaxis in AAV patients treated with rituximab as a remission-induction regimen.^9^ This may be explained by the fact that besides its action on *P. jirovecii*, TMP/SMX is also active on bacteria. TMP/SMX is commonly used to prevent *P. jirovecii* infections and has been recommended by the European League Against Rheumatism (EULAR) and the European Renal Association−European Dialysis and Transplant Association (ERA-EDTA) for patients treated with cyclophosphamide.S6 However, no formal recommendation is given on how long it should be maintained.

We are able to confirm that severe infections are very common in ANCA-GN patients,^2^^,^^5^ corroborating a recent multicenter retrospective study showing a higher incidence in these patients as compared to an age- and sex-matched population.^2^ The high infection risk at ANCA-GN diagnosis may be explained by different commonly admitted factors such as high vasculitis activity and a heavy immunosuppressive drug regimen. Previous studies, not specifically conducted in ANCA-GN patients, have identified older age, impaired kidney function, and dialysis dependency at diagnosis as constant risk factors for infection,^4^^,^^5^^,^^9^ which we also found in our study.

In recent large trials (MEPEX and PEXIVAS trials),S7,S8 infections represented a major risk factor for death in ANCA-GN patients. Our observation that kidney function at month 6 (and not at ANCA-GN diagnosis) is the major risk factor for late infection suggests that infection risk may decrease if kidney function improves following remission-induction treatment. Thus, early ANCA-GN diagnosis and prompt initiation of immunosuppressive treatments, by maximizing the changes in renal recovery, appear as to be major factors in the reduction of infection risk.

It is important, and in line with recent prospective trials, that we did not observe any difference in infection risk according to type of immunosuppressive drug regimen.S9,S10 A previous retrospective study also showed that maintaining a low dose of steroids after 6 months was associated with an increased infection risk.^6^ Despite infection risk tending to be higher in patients maintaining steroids at 6 months in our study, it did not reach statistical significance. In opposition to some past studies showing an increased rate of severe infection in patients with ear−nose−throat or pulmonary (i.e., endobronchial stenosis) involvement,^9^^,^S4 we did not observe any impact of extrarenal AAV involvement or of ANCA subtype on infection risk. These discrepancies may be related to the selection of ANCA-GN patients with severe renal impairment in our study.

The limitations of our study must be underlined: the observational and retrospective design, and inclusions covering a 20-year period with substantial modifications in therapeutic strategies. Moreover, vaccination status against influenza and pneumococcus were not available, and their frequencies may have increased over time. Finally, ICU admission was identified as a risk factor for early infection; however we cannot exclude the possibility that infection was in fact the cause of ICU admission at least in some patients.

In conclusion, our study, performed in a large and well-characterized cohort of ANCA-GN patients,S11,S12 displaying an incidence of serious infectious events in line with the current literature, identifies different risk factors over time. Early infections were associated with older age and severity of the vasculitis, or the presence of high comorbidity (ICU requirement), whereas late infections were instead associated with kidney function. Importantly, we found that long-term prophylaxis with TMP/SMX was associated with a decreased risk of mainly late infections. These data open new perspectives for infection prevention in ANCA-GN, suggesting a further focus in prospective studies on the long-term use of TMP/SMX in ANCA-GN patients.

## Disclosures

All the authors declare no competing interests.

## Supplementary Material

Supplementary File (PDF)

**Supplementary References**

**Supplementary Methods**

**Table S1.** Baseline characteristics of the population and main outcomes

**Table S2.** Nature of infections observed in the cohort

**Table S3.** Detail of bacteria isolated

**Table S4.** Univariable cox analysis of risk factors associated with first infectious event

**Figure S1.** Number of patients according to number of infectious episodes during follow-up

**Figure S2.** Survival free of severe bacterial infection (A), viral infection (B), and fungal infection (C) according to trimethoprim-sulfamethoxazole prophylaxis (B).
