# Supplementary material for: Incidence and Temporal Trend in Risk Factors of Severe Infections in ANCA-Glomerulonephritis Patients
Source: Kidney Int Rep. 2021 Jan 7;6(4):1161–5. doi: 10.1016/j.ekir.2020.12.037 (PMC8071615; doi:10.1016/j.ekir.2020.12.037)

SUPPLEMENTARY MATERIAL.

Supplementary References.

Supplementary Methods.

Supplementary Tables.

Table S1. Baseline characteristics of the population and main outcomes.

Table S2. Nature of infections observed in the cohort.

Table S3. Detail of bacteria isolated.

Table S4. Univariable cox analysis of risk factors associated with first infectious event.

Supplementary Figures.

Supplemental Figure 1. Number of patients according to number of infectious episodes during follow-up.

Supplemental Figure 2. Survival free of severe bacterial infection (A), viral infection (B) and fungal infection (C) according to trimethoprim-sulfamethoxazole prophylaxis (B).

SUPPLEMENTARY REFERENCES.

S1. Flossmann O, Berden A, de Groot K, Hagen C, Harper L, Heijl C, Hoglund P, Jayne D, Luqmani R, Mahr A, Mukhtyar C, Pusey C, Rasmussen N, Stegeman C, Walsh M, Westman K, European Vasculitis Study G: Long-term patient survival in ANCA-associated vasculitis. Annals of the rheumatic diseases 70:488-94, 2011

S2. Frohnert PP, Sheps SG: Long-term follow-up study of periarteritis nodosa. Am J Med 43:8-14, 1967

S3. Shi YY, Li ZY, Zhao MH, Chen M: The CD4 Lymphocyte Count is a Better Predictor of Overall Infection Than the Total Lymphocyte Count in ANCA-Associated Vasculitis Under a Corticosteroid and Cyclophosphamide Regimen: A Retrospective Cohort. Medicine (Baltimore) 94:e843, 2015

S4. Yoo J, Jung SM, Song JJ, Park YB, Lee SW: Birmingham vasculitis activity and chest manifestation at diagnosis can predict hospitalised infection in ANCA-associated vasculitis. Clin Rheumatol 37:2133-41, 2018

S15. Mohammad AJ, Segelmark M, Smith R, Englund M, Nilsson J-Å, Westman K, Merkel PA, Jayne DRW. Severe Infection in Antineutrophil Cytoplasmic Antibody-associated Vasculitis. J Rheumatol 44:1468–1475, 2017

S6. Yates M, Watts RA, Bajema IM, Cid MC, Crestani B, Hauser T, Hellmich B, Holle JU, Laudien M, Little MA, Luqmani RA, Mahr A, Merkel PA, Mills J, Mooney J, Segelmark M, Tesar V, Westman K, Vaglio A, Yalcindag N, Jayne DR, Mukhtyar C: EULAR/ERA-EDTA recommendations for the management of ANCA-associated vasculitis. Annals of the rheumatic diseases 75:1583-94, 2016

S7. Walsh M, Merkel PA, Peh CA, Szpirt WM, Puechal X, Fujimoto S, Hawley CM, Khalidi N, Flossmann O, Wald R, Girard LP, Levin A, Gregorini G, Harper L, Clark WF, Pagnoux C, Specks U, Smyth L, Tesar V, Ito-Ihara T, de Zoysa JR, Szczeklik W, Flores-Suarez LF, Carette S, Guillevin L, Pusey CD, Casian AL, Brezina B, Mazzetti A, McAlear CA, Broadhurst E, Reidlinger D, Mehta S, Ives N, Jayne DRW, Investigators P: Plasma Exchange and Glucocorticoids in Severe ANCA-Associated Vasculitis. N Engl J Med 382:622-31, 2020

S8. Jayne DR, Gaskin G, Rasmussen N, Abramowicz D, Ferrario F, Guillevin L, Mirapeix E, Savage CO, Sinico RA, Stegeman CA, Westman KW, van der Woude FJ, de Lind van Wijngaarden RA, Pusey CD, European Vasculitis Study G: Randomized trial of plasma exchange or high-dosage methylprednisolone as adjunctive therapy for severe renal vasculitis. J Am Soc Nephrol 18:2180-8, 2007

S9. Jones RB, Tervaert JW, Hauser T, Luqmani R, Morgan MD, Peh CA, Savage CO, Segelmark M, Tesar V, van Paassen P, Walsh D, Walsh M, Westman K, Jayne DR, European Vasculitis Study G: Rituximab versus cyclophosphamide in ANCA-associated renal vasculitis. N Engl J Med 363:211-20, 2010

S10. Stone JH, Merkel PA, Spiera R, Seo P, Langford CA, Hoffman GS, Kallenberg CG, St Clair EW, Turkiewicz A, Tchao NK, Webber L, Ding L, Sejismundo LP, Mieras K, Weitzenkamp D, Ikle D, Seyfert-Margolis V, Mueller M, Brunetta P, Allen NB, Fervenza FC, Geetha D, Keogh KA, Kissin EY, Monach PA, Peikert T, Stegeman C, Ytterberg SR, Specks U, Group R-IR: Rituximab versus cyclophosphamide for ANCA-associated vasculitis. N Engl J Med 363:221-32, 2010

S11. Guibert F, Garnier AS, Wacrenier S, Piccoli G, Djema A, Gansey R, Demiselle J, Brilland B, Cousin M, Besson V, Duveau A, El Nasser K, Coindre JP, Croue A, Saint-Andre JP, Chevailler A, Subra JF, Augusto JF: Patients with ANCA-Associated Glomerulonephritis and Connective Tissue Diseases: A Comparative Study from the Maine-Anjou AAV Registry. J Clin Med 8:2019

S12. Henry N, Brilland B, Wacrenier S, Djema A, Garnier AS, Gansey R, Coindre JP, Besson V, Duveau A, Subra JF, Cousin M, Piccoli GB, Augusto JF: Incidence and Risk Factors of Venous Thromboembolic Events in Patients with ANCA-Glomerulonephritis: A Cohort Study from the Maine-Anjou Registry. J Clin Med 9:2020

S13. Jennette JC, Falk RJ, Bacon PA, Basu N, Cid MC, Ferrario F, Flores-Suarez LF, Gross WL, Guillevin L, Hagen EC, Hoffman GS, Jayne DR, Kallenberg CG, Lamprecht P, Langford CA, Luqmani RA, Mahr AD, Matteson EL, Merkel PA, Ozen S, Pusey CD, Rasmussen N, Rees AJ, Scott DG, Specks U, Stone JH, Takahashi K, Watts RA: 2012 revised International Chapel Hill Consensus Conference Nomenclature of Vasculitides. Arthritis Rheum 65:1-11, 2013

S14. Luqmani RA, Bacon PA, Moots RJ, Janssen BA, Pall A, Emery P, Savage C, Adu D: Birmingham Vasculitis Activity Score (BVAS) in systemic necrotizing vasculitis. QJM : monthly journal of the Association of Physicians 87:671-8, 1994

S15. Levey AS, Bosch JP, Lewis JB, Greene T, Rogers N, Roth D: A more accurate method to estimate glomerular filtration rate from serum creatinine: a new prediction equation. Modification of Diet in Renal Disease Study Group. Ann Intern Med 130:461-70, 1999

SUPPLEMENTARY METHODS

Maine-Anjou Registry.

The Maine-Anjou registry is a multicenter database which began on 01/01/2018. It includes data from all adult patients with ANCA-GN diagnosed since 01/01/2000 in the Nephrology units of 4 hospitals (Angers University Hospital and the Regional Hospitals of Le Mans, Cholet and Laval). Patients included in the registry are at least 18 years old, fulfill Chapel Hill Consensus Conference criteria for AAV[S13]. They have presumed (active urinary sediment, proteinuria, and/or impaired renal function, associated with ANCA positivity) or histologically confirmed renal involvement of AAV. The registry collects data concerning presentation at ANCA-GN diagnosis (clinical and biological data), treatment, and outcomes. Data were collected retrospectively at the registry creation in 2018, and then prospectively every 6 months. The registry has been authorized by the "Commission National Informatique et Liberté" (CNIL, agreement number 2018-MR03-02). In accordance with French law, participants gave their non-opposition to be included in the registry and for the use of their data anonymously. The present study was approved by the local ethic committee of Angers University Hospital (CE 2020/84).

Data collection and definitions.

For the present study, the following data were retrieved: age, gender, weight, height, body mass index (BMI), comorbidities (hypertension and diabetes mellitus), and organ involvement at AAV diagnosis. The AAV activity was determined using the Birmingham Vasculitis Activity Score (BVAS) 2003[S14]. Medications and biological parameters at AAV diagnosis were retrieved.

The date of AAV diagnosis was defined as the day of start of remission-induction treatment (initiation of high dose methylprednisolone administration, or oral steroid therapy, usually 1

mg/kg/day). Patients were followed from ANCA-GN diagnosis until last clinical follow-up or death. Patients that were lost to follow-up before 3 months were excluded from the study. The estimated glomerular filtration rate (eGFR) was calculated using the 4-variable Modification of Diet in Renal Disease (MDRD) study equation[S15]. Patients on dialysis were considered to have an eGFR of 5 mL/min/1.73 m^2. End-stage renal disease was defined as the need for kidney replacement therapy for more than 3 months or kidney transplantation.

Severe infection was defined as bacterial, viral, fungal or parasitic infection that required hospital admission. Early infections were defined as infectious events that occurred within the 6 first months following the start of remission-induction immunosuppressive treatment and late infections as events that developed after 6 months. This cut-off have been selected as previously described[8,S5]. Moreover, it is the approximate duration of the remission induction regimen.

Prophylaxis with TMP/SMX consisted in 800/160 mg on alternate days, as recommended in the EULAR/ERA-EDTA guidelines[S6].

Statistical analysis.

Continuous variables are presented as median and interquartile range. Categorical variables are presented as effective and percentage. Differences between groups were analyzed using the χ^2 test (or Fisher exact test when applicable) for categorical variables and the Mann-Whitney U test for continuous variables. Rate of infection was expressed in reference to 100 person-years. The Kaplan-Meyer method was used to estimate patient's survival and infection free survival. A log-rank test was used to compare the survival curves. An univariable cox proportional hazards regression analysis was performed to examine factors associated with the occurrence of an initial infectious event and are reported as hazard ratio (HR) with 95% CIs.

A multivariable cox regression was carried out by including all the significant variables at threshold 0.1. Two supplemental cox analyses were performed to analyze the factors associated with 1) early infection and 2) late infections.

All the statistical tests were performed to the two-sided 0.05 level of significance. Statistical analysis was performed using SPSS software® 23.0 and GraphPad Prism®.

SUPPLEMENTARY TABLES LEGENDS

Table S1. Baseline characteristics of the population and main outcomes.

Data are presented as median and 25-75 percentile for continuous variables and absolute value and percentage for categorical variables.

*157 patients were started with maintenance regimen

ANCA, anti-neutrophil cytoplasmic antibodies; BMI, body mass index; GN, glomerulonephritis; ICU, intensive care unit; BVAS, Birmingham Vasculitis Activity Index; MPO, myeloperoxidase; eGFR, estimated glomerular filtration rate; TMP/SMX, trimethoprim sulfamethoxazole.

Table S2. Nature of infections observed in the cohort.

Table S3. Detail of bacteria isolated.

Table S4. Univariable cox analysis of risk factors associated with first infectious event.

* Per 10 mL/min/1.73m2 increment.

** Per 10 mg/L increment, data available in 150 patients, excluded from multivariable model

*** Data available in 129 patients, excluded from multivariable model.

**** Data available in 119 patients.

ANCA, anti-neutrophil cytoplasmic antibodies; BMI, body mass index; GN, glomerulonephritis; ICU, intensive care unit; BVAS, Birmingham Vasculitis Activity Index; MPO, myeloperoxidase; eGFR, estimated glomerular filtration rate; TMP/SMX, trimethoprim-sulfamethoxazole.

Table S1.

	All, n=168
**Baseline characteristics**	
Gender, M/F	104/64
Age, years	68.0 [57.0-74.0]
BMI, Kg/m^2	25.0 [22.4-28.1]
Hypertension, n (%)	91 (54.2)
Diabetes mellitus, n (%)	23 (13.7)
**ANCA-associated vasculitis characteristics, n (%)**	
Newly diagnosed	159 (94.6)
Admission to ICU at ANCA-GN diagnosis	16 (9.5)
BVAS at AAV diagnosis or relapse	16 [12-20]
ANCA subtype, n (%)	
PR3 ANCA	52 (31.0)
MPO ANCA	114 (67.9)
ANCA negative	2 (1.2)
Organ involvement at onset	
Cutaneous signs, n (%)	29 (17.3)
Ear, nose, throat, n (%)	60 (35.7)
Heart, n (%)	10 (6.0)
Digestive, n (%)	9 (5.4)
Lung, n (%)	67 (39.9)
Neurological, n (%)	24 (14.3)
Renal	
Serum creatinine, µmol/L	250 [124-417]
eGFR, mL/min/1.73 m^2	18.1 [9-40]
Proteinuria, g/g	1.57 [0.79-2.91]
Need for kidney replacement therapy, n (%)	42 (25)
**Remission-Induction regimen**, n (%)	
Cyclophosphamide	139 (87.7)
Rituximab	15 (8.9)
Other	14 (8.3)
Methylprednisolone pulses	134 (79.8)
Plasma exchange	46 (27.4)
TMP/SMX prophylaxis	108 (63.2)
**Maintenance treatment, n (%)***	
Azathioprine	83 (52.9)
Rituximab	50 (31.8)
Other	24 (15.3)
**Outcomes**, n (%)	
End-stage renal disease	46 (27.4)
Death	43 (25.6)
**Follow-up** (months)	47.9 [18.4-93.7]

Table S2.

	Number of events n (%)	Number of patients n (%)
**Overall infections**	**235**	**90 (53.6)**
**Bacterial infections**	**187 (79.6)**	**78 (46.4)**
Site of infection		
Pulmonary	76 (40.6)	42 (53.8)
Urinary tract	39 (20.9)	24 (30.8)
Gastro-intestinal tract	22 (11.8)	18 (23.1)
Skin	18 (9.6)	13 (16.6)
Articular	7 (3.7)	4 (5.1)
Other sites or undetermined	25 (13.4)	21 (26.9)
**Viral infections**	**28 (11.9)**	**22 (13.1)**
CMV	7 (25.0)	5 (22.7)
Influenzae	5 (17.9)	5 (22.7)
Herpes viruses	7 (25.0)	7 (31.8)
Other viruses	5 (17.9)	4 (18.2)
Undetermined	4 (14.3)	3 (13.6)
**Fungal or parasitic infection**	**20 (8.5)**	**16 (9.5)**
Pneumocystis Jiroveci	8 (40.0)	7 (43.8)
Aspergillus fumigatus	5 (25.0)	4 (25.0)
Candida species	4 (20.0)	4 (25.0)
Other	3 (15.0)	3 (18.8)

Table S3.

Isolated bacteria	n (%)
**Episodes with microbiological isolates**	**105**
**Gram negative bacteria**	**62 (59.0)**
Escherichia coli	24 (38.7)
Pseudomonas	11 (17.7)
Enterobacter cloacae	4 (6.5)
Other Enterobacteriaceae	13 (21.0)
Anaerobic	1 (1.6)
Coccobacillus	9 (14.5)
**Gram positive bacteria**	**28 (26.7)**
Streptococcus pneumoniae	4 (14.3)
Viridans group streptococci	4 (14.3)
Staphylococcus aureus	5 (17.9)
Coagulase-negative *staphylococcus*	9 (32.1)
Enterococcus	3 (10.7)
Anaerobic	2 (7.1)
Gram positive bacillus	1 (3.6)
**Mycobacteria**	**4 (3.8)**
**Polymicrobial**	**11 (10.5)**

Table S4.

Univariate analysis of risk factors for severe infections	All infections			Early infections			Late infections		
	HR	IC	P	HR	IC	P	HR	IC	P
**Baseline characteristics at ANCA-GN diagnosis**									
Gender (Male)	1.44	0.91-2.28	0.115	1.11	0.63-1.95	0.714	1.58	0.87-2.84	0.130
Age (years)	1.04	1.02-1.06	**<0.001**	1.04	1.01-1.06	**0.002**	1.03	1.01-1.05	**0.013**
BMI (Kg/m^2)	1.02	0.96-1.09	0.454	0.97	0.90-1.05	0.416	1.07	0.99-1.16	0.104
Hypertension	1.28	0.84-1.95	0.245	1.57	0.89-2.76	0.118	0.99	0.59-1.67	0.987
Diabetes mellitus	1.16	0.66-2.06	0.602	1.14	0.54-2.42	0.733	1.27	0.64-2.51	0.498
**ANCA-associated vasculitis characteristics**									
Newly diagnosed	1.64	0.66-4.06	0.288	3.12	0.43-22.6	0.259	1.15	0.46-2.90	0.760
Admission in ICU at ANCA-GN diagnosis	3.34	1.78-6.26	**<0.001**	3.62	1.86-7.08	**<0.001**	0.84	0.26-2.73	0.777
BVAS at AAV at ANCA-GN diagnosis	1.02	0.99-1.06	0.244	1.03	0.99-1.08	0.117	0.98	0.94-1.03	0.443
MPO-ANCA or No ANCA	1.42	0.88-2.28	0.151	1.17	0.63-2.15	0.623	2.00	1.06-3.78	**0.033**
Organ involvement, n (%)									
Cutaneous signs	0.94	0.55-1.63	0.944	1.04	0.51-2.14	0.907	0.87	0.44-1.72	0.687
Ear, nose, throat	1.14	0.74-1.75	0.561	0.99	0.56-1.77	0.998	1.14	0.66-1.95	0.643
Heart	1.94	0.84-4.46	0.118	1.75	0.63-4.85	0.284	1.43	0.45-4.60	0.546
Digestive	0.20	0.48-2.97	0.692	0.71	0.17-2.90	0.628	1.31	0.41-4.21	0.652
Lung	1.24	0.82-1.90	0.313	1.45	0.84-2.51	0.178	1.02	0.60-1.73	0.949
Neurological	0.97	0.53-1.78	0.923	1.10	0.52-2.33	0.812	0.62	0.25-1.55	0.306
Kidney (at ANCA-GN diagnosis)									
eGFR, mL/min/1.73 m^2 *	0.88	0.81-0.96	**0.005**	0.90	0.80-1.01	0.070	0.86	0.77-0.96	**0.006**
Need for kidney replacement therapy	2.28	1.44-3.61	**<0.001**	2.95	1.70-5.13	**<0.001**	1.71	0.95-3.10	0.074
Renal limited vasculitis (vs systemic vasculitis)	0.87	0.52-1.46	0.603	0.53	0.24-1.17	0.115	1.25	0.67-2.32	0.480
**Biology at ANCA-GN diagnosis**									
C-reactive protein, mg/L**	1.03	1.00-1.05	**0.032**	1.03	1.00-1.06	0.580	1.01	0.98-1.04	0.377
Serum albumin, g/L	0.97	0.94-1.01	0.166	0.99	0.94-1.03	0.545	0.97	0.92-1.01	0.141
Lymphocyte count, G/L***	0.68	0.47-0.99	**0.043**	0.60	0.36-1.01	0.053	0.93	0.58-1.48	0.754
Immunoglobulin level, g/L****	0.99	0.94-1.05	0.755	0.97	0.91-1.05	0.450	0.99	0.92-1.06	0.741
**Remission-induction regimen**									
Cyclophosphamide	1.24	0.69-2.24	0.469	2.78	1.00-7.72	**0.049**	0.65	0.33-1.29	0.215
Rituximab	0.52	0.19-1.43	0.205	0.18	0.02-1.27	0.175	1.19	0.63-1.25	0.592
Methylprednisolone pulses	1.18	0.69-2.03	0.546	2.11	0.90-4.94	0.085	0.70	0.37-1.33	0.282
Plasma exchange	1.40	0.89-2.19	0.148	1.73	0.99-3.05	0.056	1.33	0.76-2.36	0.321
**Prophylaxis with TMP/SMX**	0.62	0.41-0.94	**0.025**	0.90	0.51-1.57	0.702	0.54	0.32-0.91	**0.020**
**Maintenance treatment**									
Steroids at month 6							2.95	0.41-21.4	0.294
Steroids > 10 mg/day month 6							1.10	0.62-1.95	0.745
Azathioprine							0.86	0.49-1.51	0.594
Rituximab							0.75	0.35-1.61	0.467
**Renal function**									
eGFR at month 6, mL/min/1.73 m^2 **							0.78	0.68-0.89	**<0.001**
**Infection between ANCA-GN diagnosis and months 6**							1.43	0.83-2.48	0.202

SUPPLEMENTARY FIGURES LEGENDS

Supplemental Figure 1. Number of patients according to number of infectious episodes during follow-up.

Supplemental Figure 2. Survival free of severe bacterial infection (A), viral infection (B) and fungal infection (C) according to trimethoprim-sulfamethoxazole prophylaxis (B).

Supplemental Figure 1

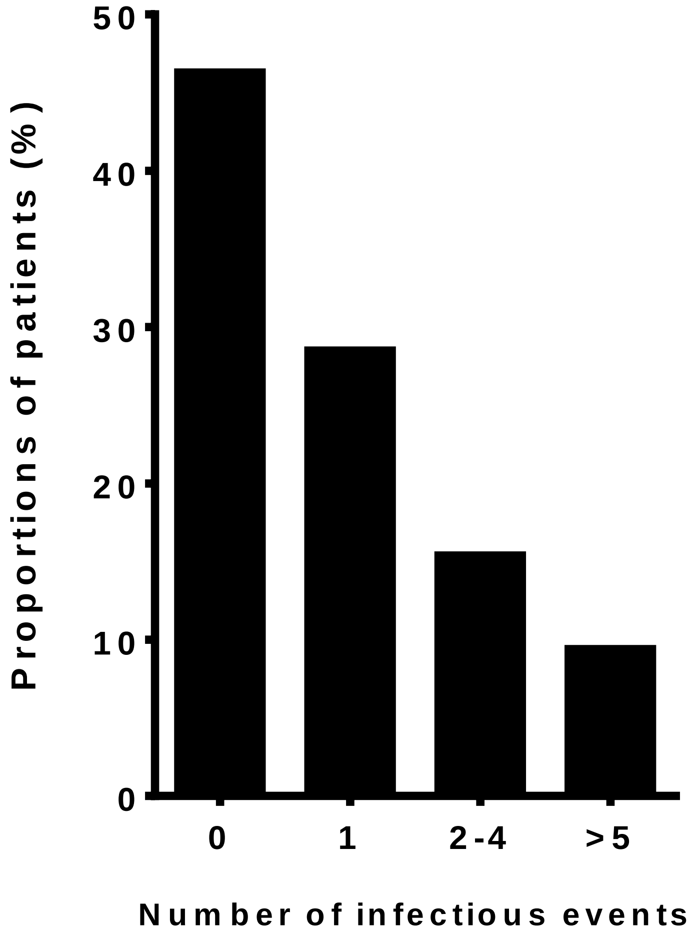

Supplemental Figure 2

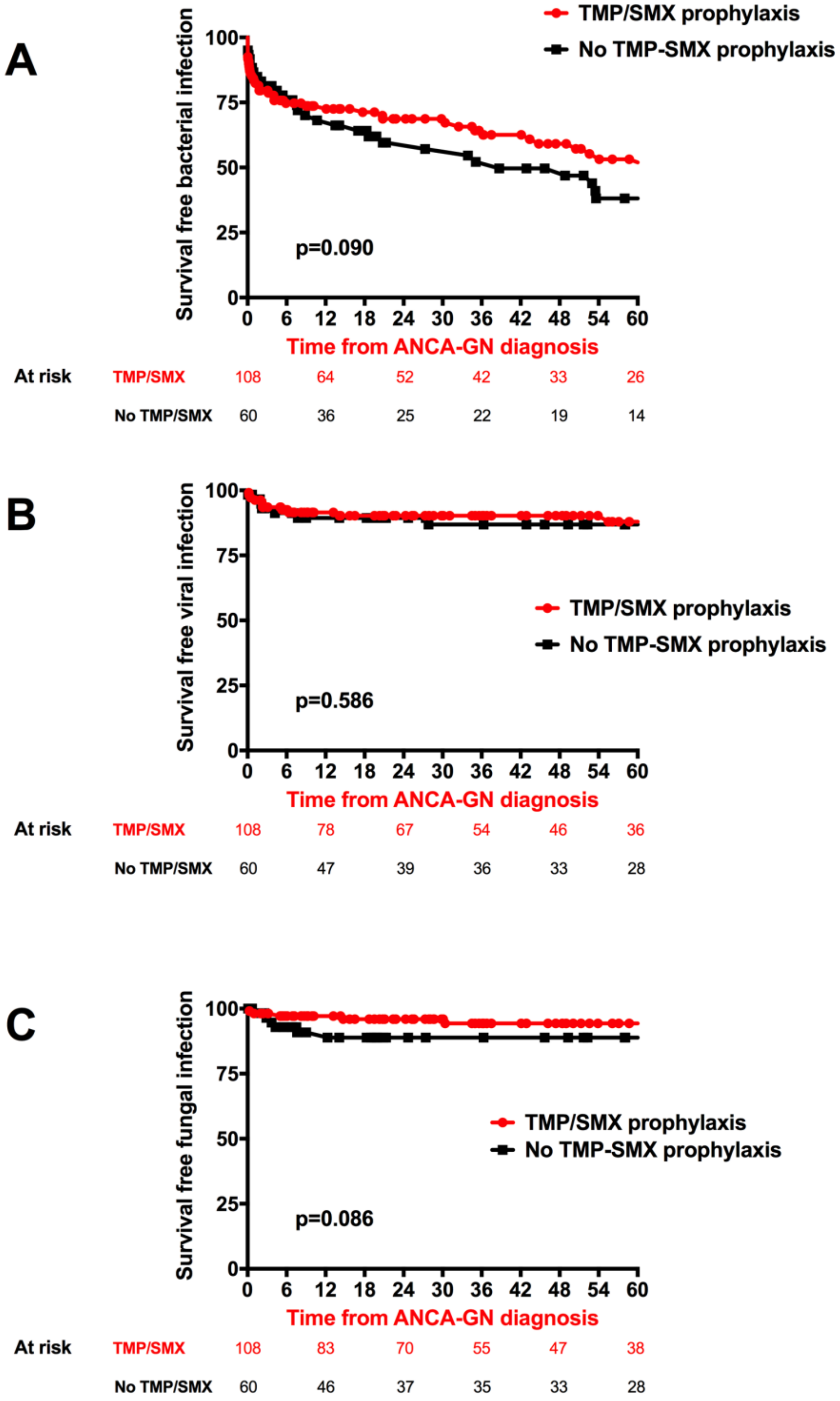

Supplement: Supplementary File (PDF) [file mmc1.pdf]
